# Antimicrobial Susceptibility Profiles of *Escherichia coli* Isolates from Clinical Cases of Geese in Hungary Between 2022 and 2023

**DOI:** 10.3390/antibiotics14050450

**Published:** 2025-04-29

**Authors:** Ádám Kerek, Ábel Szabó, Ákos Jerzsele

**Affiliations:** 1 Department of Pharmacology and Toxicology, University of Veterinary Medicine Budapest, István utca 2, HU-1078 Budapest, Hungary; szabo.abel@student.univet.hu (Á.S.); jerzsele.akos@univet.hu (Á.J.); 2 National Laboratory of Infectious Animal Diseases, Antimicrobial Resistance, Veterinary Public Health and Food Chain Safety, University of Veterinary Medicine Budapest, István utca 2, HU-1078 Budapest, Hungary

**Keywords:** *Escherichia coli*, antimicrobial resistance, minimum inhibitory concentration, MIC, waterfowl, geese

## Abstract

**Background**: Antimicrobial resistance (AMR) poses an increasing threat to animal health and food safety. In the poultry sector, particularly in waterfowl farming, the widespread use of antibiotics may contribute to the dissemination of resistant *Escherichia coli* strains. This study aims to map the antibiotic resistance profiles of *E. coli* isolates from geese in Hungary, determine the prevalence of multidrug-resistant (MDR) and extensively drug-resistant (XDR) strains, and analyze resistance patterns and co-resistance relationships. **Methods**: *E. coli* isolates from clinical cases between 2022 and 2023 were examined using minimum inhibitory concentration (MIC) determination. Susceptibility results were evaluated based on the Clinical Laboratory Standard Institute (CLSI) breakpoints. Cluster analysis and principal component analysis (PCA) were applied to identify resistance patterns. Co-resistance relationships were examined through network analysis, while Monte Carlo simulations were used to estimate the expected prevalence of MDR strains. **Results**: Among the examined isolates, neomycin resistance was particularly high (86.8%), while florfenicol (73.6%) and amoxicillin (65.9%) resistance levels were also significant. The prevalence of MDR strains was 86.8%, and XDR strains accounted for 38.5%. Co-resistance analysis revealed a strong correlation between neomycin and spectinomycin resistance, as well as amoxicillin and doxycycline resistance. Monte Carlo simulations estimated that the expected range of MDR strain prevalence could vary between 80.2% and 92.3%. **Conclusions**: The high prevalence of MDR and XDR strains highlights the urgent need to reassess antibiotic usage strategies in goose farming. These findings underscore the importance of targeted antibiotic use, continuous microbiological surveillance, and the exploration of alternative therapeutic approaches to mitigate AMR.

## 1. Introduction

Zoonotic infections have accompanied human history, with estimates suggesting that nearly 60% of known human infections originate from animals. Zoonotic pathogens include viruses, parasites, and fungi [1], but bacterial infections account for approximately 42% of zoonotic cases [2]. Antimicrobial resistance (AMR), particularly the spread of multidrug-resistant (MDR) pathogens, poses an increasing challenge to both human and veterinary medicine worldwide [3].

Bacteria that are part of the gut microbiome are widespread in nature, with both pathogenic and non-pathogenic strains present [4]. *Escherichia coli* is one of the most common and significant species, typically found as a commensal microorganism in the intestines of humans and animals. However, some pathotypes can cause gastrointestinal diseases, urinary tract infections, and bacteremia [5]. Beyond its pathogenic potential, *E. coli* is also widely used as an indicator species for monitoring antibiotic resistance [6]. Over the past years, MDR *E. coli* strains have increasingly posed a serious issue in both human and veterinary medicine [3].

Among various environmental reservoirs, birds play a crucial role in the spread of antibiotic resistance, acting as vectors between humans and other animal species [7]. Several studies have demonstrated that poultry can serve as both reservoirs and disseminators of resistant bacteria and antimicrobial resistance genes (ARGs), particularly in *E. coli* [8,9]. Domestic animals and poultry flocks have become significant sources of antibiotic-resistant bacteria for human populations [2,10,11].

Excessive antibiotic use and the low absorption of orally administered drugs result in a substantial proportion of antibiotics being excreted into the environment, maintaining a constant selection pressure on pathogenic bacteria such as *E.coli* [12,13]. As a consequence, resistant bacteria and ARGs spread rapidly, posing a threat to both ecosystems and human health [14]. To address this issue, alternative strategies are necessary to partially or completely replace antibiotics [15], including plant-derived antibacterial compounds [16,17,18], antimicrobial peptides [19], medium-chain fatty acids and nanometals [20,21], and probiotics and prebiotics [22]. Additionally, biosecurity measures on farms [23] and pre-treatment pharmacological evaluations of antibiotics are essential for mitigating resistance development [24].

Geese are among the oldest domesticated poultry species, with two major centers of domestication: the Chinese domestic goose over 7000 years ago [25], and the European domestic goose approximately 5000 years ago [26]. Today, goose farming remains an important industry, providing nutrient-rich meat. However, the presence of antibiotic residues and resistant bacterial strains has raised increasing concerns in the industry.

The emergence of MDR *E. coli* strains complicates the treatment of bacterial infections and poses a public health risk. The rapid spread of antibiotic resistance among bacterial isolates has become a significant and growing concern, affecting both animal and human health [27].

In this study, we examined the antibiotic resistance profiles of *E. coli* isolates from clinical cases in geese to gain a better understanding of antimicrobial resistance in goose farming and contribute to the development of veterinary strategies and antibiotic usage guidelines.

## 2. Results

A total of 91 *E. coli* strains isolated from clinical cases were subjected to phenotypic antimicrobial susceptibility testing, with minimum inhibitory concentration (MIC) determination. The majority of isolates originated from the Dél-Alföld region of Hungary (59.3%; *n* = 54), followed by the Észak-Magyarország region (20.9%; *n* = 19), the Észak-Alföld region (14.3%; *n* = 13), the Közép-Magyarország region (3.3%; *n* = 3), and the Közép-Dunántúl region (2.2%; *n* = 2).

**Figure 1:**
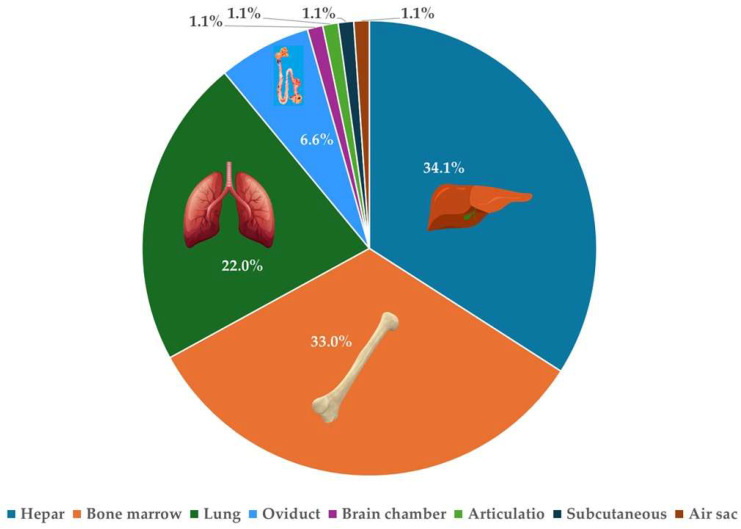
The anatomical distribution of *Escherichia coli* isolates (*n* = 91) and their percentage representation.

The isolates were obtained from various organ samples (Figure 1), with the liver being the most frequent source (*n* = 31), followed by bone marrow (*n* = 30), lungs (*n* = 20), and oviduct (*n* = 6). Additionally, single isolates were obtained from the brain ventricle, joint, subcutaneous tissue, and air sacs.

**Figure 2:**
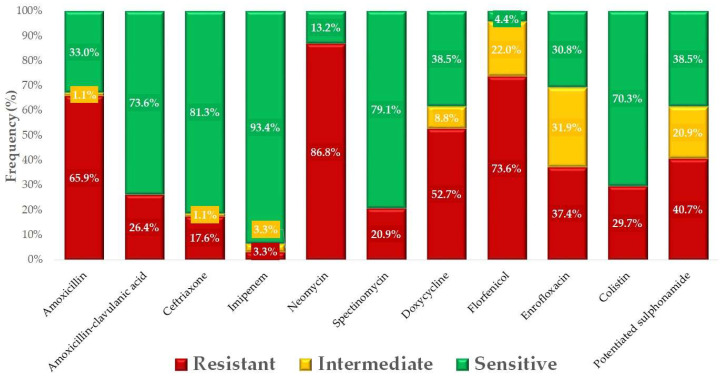
Antimicrobial susceptibility profile of *Escherichia coli* isolates (*n* = 91) from geese, tested against clinically and public health-relevant antibiotics.

Using clinical breakpoints, we determined the proportion of isolates classified as susceptible, intermediate, or resistant to each antibiotic (Figure 2). The majority of isolates (86.8%) were resistant to neomycin, while high resistance rates were also observed for florfenicol (73.6%), colistin (29.7%), and enrofloxacin (37.4%). The lowest resistance rate was recorded for imipenem (3.3%), with only 3.3% of isolates showing reduced susceptibility to this antibiotic.

**Figure 3:**
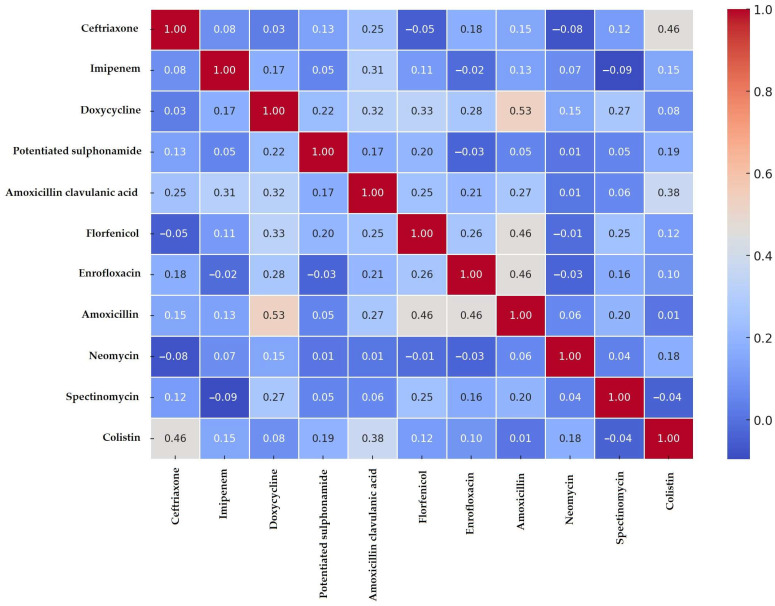
Correlation analysis of antimicrobial resistance patterns in *Escherichia coli* isolates, visualized as a heatmap.

Based on the resistance profiles determined using clinical breakpoints, a correlation analysis (Figure 3) was performed to identify potential associations between resistance patterns of different antibiotics. The analysis revealed strong positive correlations, particularly between amoxicillin and doxycycline (0.53), amoxicillin and florfenicol (0.46), amoxicillin and enrofloxacin (0.46), as well as colistin and ceftriaxone (0.46). These findings suggest that co-resistance mechanisms may contribute to the co-selection of resistance among these antibiotic classes, potentially affecting treatment efficacy and promoting the spread of multidrug-resistant strains.

The proportion of MDR strains was determined to be 86.8% (*n* = 79), meaning these isolates showed resistance to at least three antibiotics. Additionally, 38.5% (*n* = 35) of isolates were classified as extensively drug-resistant (XDR), indicating that these strains were non-susceptible to at least one agent in all but two or fewer antimicrobial categories. No pan-drug-resistant (PDR) strains were identified.

The MIC (Additional data) frequency distribution for each antibiotic is summarized in Table 1, while MIC distributions for antibiotics without clinical breakpoints are detailed in Supplementary Table S1. Our findings indicate that, based on clinical breakpoints, at least 50% of the examined isolates remained susceptible to amoxicillin–clavulanic acid, ceftriaxone, colistin, enrofloxacin, imipenem, and spectinomycin. However, 90% of isolates were only susceptible to imipenem.

**Figure 4:**
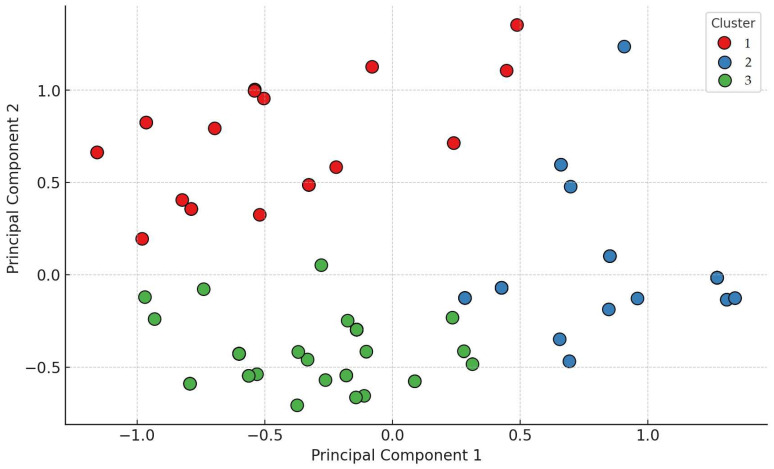
Cluster analysis based on resistance patterns, classifying isolates into three distinct clusters. Cluster 1 is marked in red, Cluster 2 in blue, and Cluster 3 in green.

A cluster analysis was performed based on resistance patterns (Figure 4). Three main clusters were identified. Cluster 1 (red) was characterized by high resistance to amoxicillin (94.9%). Cluster 2 (blue) showed notable resistance to neomycin (82.1%) and florfenicol (35.7%). Cluster 3 (green) consisted predominantly of strains resistant to potentiated sulfonamides.

When evaluated against epidemiological cut-off values (ECOFF) defined by the European Committee on Antimicrobial Susceptibility Testing (EUCAST), at least 50% of isolates were classified as wild-type for ceftriaxone. The proportion of non-wild-type strains for each antibiotic is presented in Supplementary Figure S1.

All exact MIC values and additional details on individual isolates are available in the Supplementary Materials.

**Figure 5:**
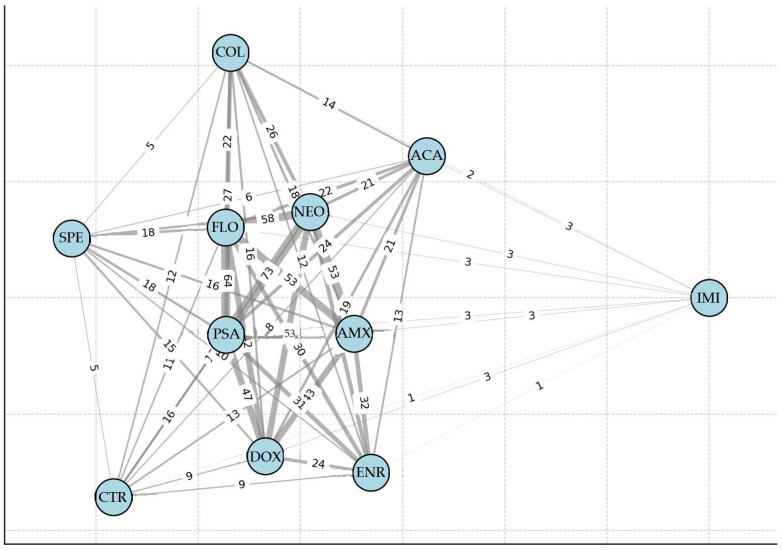
Network analysis illustrating resistance interactions using graphical models. Imipenem-resistant strains formed a distinct subgroup. Abbreviations: AMX—amoxicillin; ACA—amoxicillin–clavulanic acid; CTR—ceftriaxone; COL—colistin; DOX—doxycycline; ENR—enrofloxacin; FLO—florfenicol; IMI—imipenem; NEO—neomycin; PSA—potentiated sulfonamide; SPE—spectinomycin.

A network analysis was conducted to visualize resistance interactions (Figure 5). The results revealed that neomycin and spectinomycin resistance frequently co-occurred at high levels. Additionally, strong associations were observed between doxycycline and amoxicillin, as well as florfenicol and colistin resistance. Imipenem-resistant strains formed a distinct subgroup, suggesting unique resistance mechanisms. The strongest association with other antibiotics was observed for potentiated sulfonamides.

**Figure 6:**
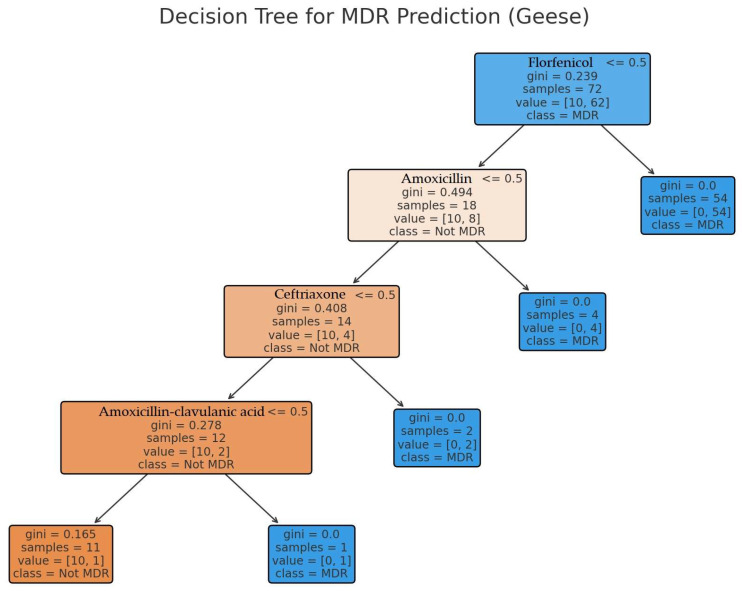
Decision tree model predicting multidrug-resistant strain occurrence. Florfenicol was selected as the primary variable due to its strong association with other antibiotics.

A predictive model was applied to forecast the occurrence of MDR strains (Figure 6). Florfenicol was selected as the primary variable, as it exhibited the strongest associations with other antibiotics in the network analysis. The model achieved 100% accuracy (precision, recall, and F1-score) in classifying MDR strains. The key decision points in the model indicate that florfenicol resistance strongly influences co-resistance to amoxicillin, ceftriaxone, and amoxicillin–clavulanic acid.

**Figure 7:**
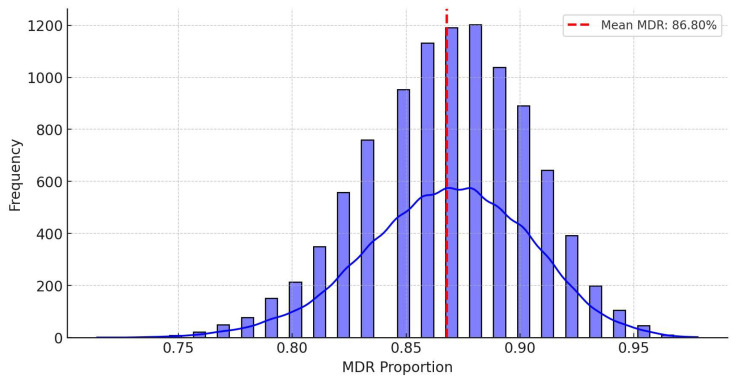
Monte Carlo simulation-based stochastic modeling to predict multidrug-resistant strain prevalence.

Stochastic modeling was subsequently performed using Monte Carlo simulations to estimate potential changes in MDR prevalence under different antibiotic usage trends (Figure 7). Through random sampling-based simulations, possible MDR prevalence rates were projected over thousands of iterations. The results indicated that the average MDR prevalence was 86.8%, with a standard deviation of 3.6%. The median and mean values coincided, suggesting a symmetrical distribution. The predicted MDR prevalence was estimated to range between 80.0% and 92.0%, with a 95% confidence interval of 80.2–92.3%.

**Figure 8:**
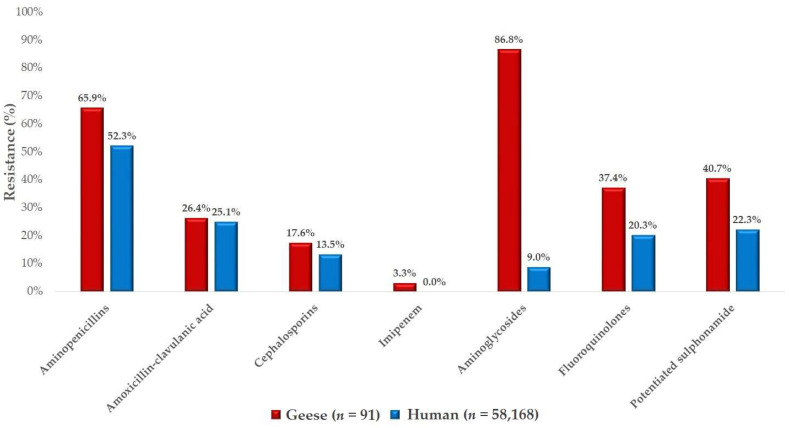
Comparison of resistance rates between *Escherichia coli* isolates from geese and human clinical isolates provided by the National Public Health and Pharmaceutical Center.

A comparative analysis was performed using human resistance data (Figure 8). The resistance patterns for aminopenicillins, amoxicillin–clavulanic acid, and ceftriaxone were found to be highly similar between human and veterinary isolates. However, aminoglycoside resistance was substantially higher in veterinary isolates, which may be attributed to their widespread use in poultry production.

## 3. Discussion

A total of 91 *E. coli* strains of goose origin were analyzed for antimicrobial susceptibility, revealing varying degrees of resistance to the tested antibiotics. The findings demonstrated that over 70% of the isolates were resistant to neomycin and florfenicol, while more than 50% showed resistance to amoxicillin and doxycycline.

*E. coli* is prone to developing resistance to most antibacterial agents; hence, it is commonly used as an indicator species for monitoring AMR. The increasing resistance of *E. coli* presents a major challenge for both animal and public health, underscoring the need for new antibiotic development or the identification of alternative therapeutic solutions.

In the current study, amoxicillin resistance was found to be 65.9%, whereas Sun et al. reported a resistance rate of 84.4% in geese isolates [27]. A strong positive correlation (0.53) was observed between amoxicillin and doxycycline resistance, suggesting the potential for co-resistance between these two antibiotics.

The resistance rate for amoxicillin–clavulanic acid was 26.4%, whereas Yassin et al. [28], Varga et al. [29], and Jeong et al. [30] did not detect any resistant isolates. The significantly lower resistance rate compared to amoxicillin alone supports the hypothesis that a substantial proportion of strains produce β-lactamase enzymes, which hydrolyze the β-lactam antibiotic component while still being inhibited by clavulanic acid.

Resistance to ceftriaxone was found in 17.6% of the isolates, which is lower than the 29% reported by Afayibo et al. [31], but higher than the 11.4% reported by Yassin et al. [28]. Meanwhile, Varga et al. [29] and Jeong et al. [30] did not detect any ceftriaxone-resistant isolates. A strong correlation (0.46) was found between ceftriaxone and colistin resistance, suggesting co-occurrence of resistance mechanisms in some isolates. Given that ceftriaxone is a third-generation cephalosporin classified as a critically important antibiotic for human medicine, its use in poultry production is strictly prohibited. Although the use of ceftriaxone and other third-generation cephalosporins is prohibited in poultry farming in the European Union, including Hungary, the observed resistance may be explained by indirect factors such as environmental contamination or horizontal gene transfer via plasmids carrying extended spectrum beta-lactamases (ESBL) genes. These resistance determinants may originate from human or other animal reservoirs and spread across bacterial populations. However, it is assumed that colistin is widely administered to farmed geese, which could potentially explain the development of resistance, making this finding unfortunate but not entirely surprising.

Resistance to imipenem was observed in 3.3% of isolates, while Sun et al. reported 100% resistance in goose isolates [27]. This discrepancy may be attributed to differences in sampling locations, methodologies, or variations in antimicrobial usage practices between the studies. Imipenem is a carbapenem antibiotic strictly reserved for human medicine and is not permitted for use in food-producing animals. While the resistance rate detected in this study was low, the presence of even a small number of imipenem-resistant isolates is concerning, as this antibiotic represents a last-line treatment for severe infections in human medicine. Although carbapenems such as imipenem are not authorized for use in poultry within the European Union, a subset of isolates in our study exhibited resistance to this antibiotic. This finding was unexpected and raises important questions regarding the possible origin of these resistance determinants. One plausible explanation is the acquisition of plasmid-mediated carbapenemase genes, potentially transferred from human or environmental sources through horizontal gene transfer. The presence of such resistance in isolates from animals not directly exposed to carbapenems highlights the complexity of AMR transmission dynamics and underscores the importance of a One Health surveillance approach that includes environmental, animal, and human sectors.

Doxycycline resistance was 52.7%, while Sun et al. [27] and Cen et al. [32] reported 86.2% and 95.4%, respectively. This discrepancy may be attributed to differences in geographical regions, sampling timeframes, and local antibiotic usage patterns, which could influence the prevalence of resistant strains. The high levels of tetracycline resistance are indicative of the long-term overuse of this antibiotic class. Doxycycline resistance exhibited strong correlations with amoxicillin (0.53) and florfenicol (0.33). Since tetracyclines are poorly absorbed in the gastrointestinal tract and largely excreted in their active form, they exert strong selection pressure on environmental microbial populations.

Notably high resistance to florfenicol (73.6%) was detected, which is higher than the 62% reported by Afayibo et al. [31]. This difference may be attributed to variations in florfenicol usage practices, sampling methods, or geographic regions. While the overall high resistance rates are consistent, differences in local antimicrobial administration protocols or selective pressure exerted by other antibiotics could explain the observed discrepancies. This high resistance rate suggests excessive use of florfenicol in the poultry industry, leading to a progressive decline in its efficacy over time.

Resistance to enrofloxacin was 37.4%, compared to 100% in the study by Afayibo et al. [31], and 58.6% in the study by Jeong et al. [30], whereas Yassin et al. [28] did not detect any resistant strains. The high variability in enrofloxacin resistance across studies suggests that national and regional differences in antibiotic usage policies significantly influence resistance levels. Although fluoroquinolone resistance can develop rapidly, resistance levels tend to decline substantially when selection pressure is removed.

For colistin, 29.7% of isolates were resistant, whereas Cen et al. reported a 9.1% resistance rate [32]. Colistin is classified as a last-resort antibiotic in human medicine, and its use in livestock production is expected to be significantly reduced in the coming years. Until recently, it was believed that colistin resistance would not emerge easily; however, the discovery of plasmid-mediated *mcr* genes has facilitated its rapid global spread [33].

Resistance to potentiated sulfonamides was found in 40.7% of isolates, whereas Yassin et al. reported 97.7% [28], Jeong et al. 51.7% [30], and Varga et al. 16.7% [29]. Given that sulfonamides have been widely used in poultry production for decades, resistance to this antibiotic class remains a significant concern. According to the decision tree analysis, the strongest predictors of MDR status in geese were florfenicol, amoxicillin, ceftriaxone, and amoxicillin–clavulanic acid. Network analysis further confirmed strong associations between resistance to potentiated sulfonamides and neomycin, reflecting their position in the same antibiotic group. Additionally, high co-resistance was observed between doxycycline and amoxicillin. In contrast, imipenem resistance did not show strong associations with other antibiotics, likely due to the low overall number of resistant strains.

The observed associations between resistance phenotypes may be influenced not only by co-selection pressures but also by the physical linkage of resistance genes on mobile genetic elements, such as plasmids or transposons. Although genomic data were not available in the present study, this potential genetic basis may partly explain the strong co-occurrence patterns identified in the network analysis.

The current study focuses on mapping phenotypic resistance profiles of *E. coli* isolates against individual antimicrobial agents. Heteroresistance profiling or evaluation of antibiotic combinations was not performed. Nonetheless, we acknowledge that combination testing (e.g., checkerboard assays, time-kill studies) could offer valuable insights into potential synergistic or antagonistic effects, particularly in the context of multidrug-resistant strains. Future studies should explore such interactions to inform more effective therapeutic strategies and identify potential avenues for mitigating resistance.

Antimicrobial resistance represents a major global public health challenge, jeopardizing our ability to treat bacterial infections effectively, as highlighted by the emergence of superbugs [34]. The World Health Organization (WHO) advocates a One Health approach to tackling AMR, emphasizing the interconnections between human, animal, and environmental health [35,36]. The widespread use of antimicrobials in both human and veterinary medicine has driven the persistent rise in bacterial resistance [37,38], with severe implications for both animal and public health [39,40].

This situation poses serious consequences for public health and has a negative socioeconomic impact on affected populations [41]. Therefore, veterinarians must rigorously monitor antibiotic usage on farms to minimize the long-term use of the same drugs and mitigate the emergence of resistance.

Several resistance patterns observed in this study are also commonly reported in human clinical isolates of *E. coli*, particularly those involving ESBL-producing strains and fluoroquinolone resistance. The overlap between resistance profiles in animal and human sources has been widely documented, suggesting potential pathways for zoonotic transmission or shared environmental reservoirs. These findings underscore the importance of integrated AMR monitoring systems that link veterinary and human health data, in line with the One Health framework. Comparative analyses with national and international human AMR surveillance reports could further support risk assessment and targeted policy interventions.

The high MDR prevalence in goose-derived *E. coli* isolates is alarming and similarly high MDR rates (>90%) have been reported in other goose populations [42,43]. These findings underscore the urgent need to revise antibiotic stewardship policies in veterinary medicine. The predictive models used in this study suggest that machine learning tools could enhance the early detection of MDR strains, facilitating optimized treatment strategies and reducing the spread of resistance. Machine learning-based predictive models, including decision trees and neural networks, provide valuable insights into resistance trends. Furthermore, Monte Carlo simulations validate these findings, reinforcing the importance of proactive antimicrobial stewardship measures.

The observed differences in antimicrobial resistance rates between our study and previously published reports may reflect variations in antimicrobial usage patterns across different geographic regions. Although country and region-specific data on antibiotic consumption in geese are limited, prior studies have shown that antimicrobial usage practices in poultry production vary considerably between countries and may influence local resistance profiles [12,44]. In addition, due to the scarcity of goose-specific AMR data in scientific literature, we referenced studies involving other avian species to provide broader context. While we recognize the limitations of interspecies comparisons, these data help position our findings within the current knowledge landscape.

Based on our findings, several policy-level interventions may be warranted. These include the development of species-specific guidelines for antimicrobial use in geese, reinforcement of surveillance systems to monitor AMR trends in minor poultry species, and targeted reduction of resistance-prone drugs such as fluoroquinolones. Furthermore, awareness campaigns and education programs aimed at veterinarians and farmers could improve responsible antibiotic stewardship. These measures align with current One Health initiatives and could help mitigate the dissemination of resistant bacteria within and beyond the poultry production chain.

## 4. Materials and Methods

### 4.1. Origin of the Strains and Human Data

The *E. coli* strains examined in this study were collected between 2022 and 2023 and were isolated by staff at the National Reference Laboratory of the Hungarian National Food Chain Safety Office (NÉBIH) from deceased and clinically diagnosed geese submitted for necropsy. The necropsies were performed by poultry health specialists, while the bacterial isolation was conducted by laboratory technicians. Species identification was performed using Coliform agar (Biolab Zrt., Budapest, Hungary). The isolates were received as pure cultures, which were subsequently stored at −80 °C using the Microbank™ system (Pro-Lab Diagnostics, Richmond Hill, Canada). The bacterial isolates used in this study were not collected by the research team but were provided as pure cultures by an independent diagnostic laboratory. Each isolate was derived from a different goose, with no duplication of sampling from the same individual. All isolates used in this study were received as pure cultures from the Hungarian National Reference Laboratory for *E. coli*. The laboratory performed the primary isolation and species-level identification of the strains based on internationally accepted microbiological standards, prior to their transfer for research use. Our study included only confirmed *E. coli* isolates, provided in monoxenic (pure) culture form.

Human AMR data were provided by the Hungarian National Public Health and Medical Officer Service. These data were related to ampicillin resistance, while in veterinary cases, amoxicillin resistance was evaluated. For third-generation cephalosporins, resistance was assessed based on ceftriaxone susceptibility. Resistance to aminoglycosides was examined collectively for gentamicin, tobramycin, and amikacin, while neomycin resistance was assessed separately. Similarly, fluoroquinolone resistance was analyzed as a group, with enrofloxacin examined individually for veterinary cases. The human resistance data, including both aggregated and region-specific data, were provided in an Excel spreadsheet with the approval of the Chief Medical Officer. The dataset included percentage values for resistance rates.

For the goose-derived isolates, data were available on the source organ (liver, bone marrow, lungs, brain chamber, oviduct, articulatio, subcutaneous, air sac) and the geographical origin of the sample, which allowed classification into seven administrative regions of Hungary. The isolates originated from five regions of Hungary: Dél-Alföld (59.3%; *n* = 54), Észak-Magyarország (20.9%; *n* = 19), Észak-Alföld (14.3%; *n* = 13), Közép-Magyarország (3.3%; *n* = 3), and Közép-Dunántúl (2.2%; *n* = 2).

### 4.2. Minimum Inhibitory Concentration (MIC) Determination

The phenotypic resistance profiles of the isolates were determined by MIC testing, following the Clinical Laboratory Standards Institute (CLSI) guidelines [45]. The breakpoints were defined based on CLSI recommendations [45], while the results were also compared to the ECOFFs established by the EUCAST [46]. The literature-based breakpoints used included references for amoxicillin–clavulanate [47], neomycin [48], spectinomycin [47], and colistin [49].

Bacterial strains stored at −80 °C were resuspended in 3 mL cation-adjusted Müller-Hinton broth (CAMHB) and incubated at 37 °C for 18–24 h prior to testing. MIC determination was performed using 96-well microtiter plates (VWR International, LLC., Debrecen, Hungary). Except for the first column, all wells were filled with 90 μL of CAMHB. The stock solutions of the tested antibiotics (Merck KGaA, Darmstadt, Germany) were prepared at 1024 μg/mL according to CLSI guidelines [50].

Amoxicillin and amoxicillin–clavulanic acid were prepared in a 2:1 ratio (pH 7.2, 0.01 mol/L), while imipenem was dissolved in phosphate buffer (pH 6, 0.1 mol/L). Doxycycline, neomycin, tylosin, and vancomycin were dissolved in distilled water. Potentiated sulfonamide (trimethoprim-sulfamethoxazole) was prepared in a 1:19 ratio, with sulfamethoxazole dissolved in hot water with 2.5 mol/L NaOH, and trimethoprim dissolved in distilled water containing 0.05 mol/L HCl. Enrofloxacin was dissolved using 1 mol/L NaOH in distilled water, while florfenicol was dissolved in a mixture of 95% ethanol and distilled water.

A two-fold serial dilution was performed, starting with 180 μL of a 512 μg/mL antibiotic solution in the first column. The final column contained only CAMHB as a negative control. The bacterial suspensions were adjusted to a 0.5 McFarland standard using a nephelometer (ThermoFisher Scientific, Budapest, Hungary) and were inoculated into the microtiter plate wells at a 10 μL/well volume [45]. MIC values were determined using the Sensititre™ SWIN™ automatic MIC reader (ThermoFisher Scientific, Budapest, Hungary), and the results were analyzed using VIZION System Software v3.4 (ThermoFisher Scientific, Budapest, Hungary, 2024). The quality control strain used was *E. coli* ATCC 25922. MIC plates were prepared in-house under sterile conditions using Mueller–Hinton broth supplemented with antibiotic concentrations prepared according to CLSI recommendations. For quality control, *E. coli* ATCC 25922 was used in every batch to verify the valid MIC values. All plates were incubated under standardized conditions (35 ± 2 °C, 18–24 h). Only data validated by appropriate control strain performance were included in the final analysis.

### 4.3. Statistical Analysis

Statistical analyses were performed using R programming language (version 4.2.2) in the RStudio environment [51]. The normality of data distribution was assessed using the Shapiro–Wilk test. Non-normally distributed data were analyzed using non-parametric tests. The Kruskal–Wallis test [52] was applied to compare the median values of different sample groups, as it does not assume a normal distribution, making it suitable for analyzing variations in resistance levels across groups. Additionally, post-hoc analyses were conducted to identify specific group differences, using Mann–Whitney U test [53] and t-tests, with pairwise comparisons between all groups. To control for multiple comparisons, Bonferroni correction [54] was applied to avoid inflated *p*-values, though it is acknowledged that this correction may increase the risk of Type II errors (failing to detect true differences).

For correlation analysis between antibiotic resistance patterns, heatmap visualization was used, employing the corrplot (v0.92) and pheatmap (v1.0.12) packages.

For cluster analysis, hierarchical clustering was performed, and the results were visualized using factoextra (v1.0.7). Agglomerative hierarchical clustering was conducted using the cluster (v2.1.4) package, while dendrogram visualization was generated using dendextend (v1.16.0).

To analyze co-resistance patterns, network analysis was conducted using the igraph (v1.3.5) package for graph construction and ggraph (v2.1.0) for visualization.

For the prediction of MDR strains, a decision tree model was built using rpart (v4.1.16). Model performance was evaluated using caret (v6.0.93), and visualization of the decision tree was performed using rpart.plot (v3.1.2).

The decision tree model was chosen for its interpretability and proven applicability in antimicrobial resistance prediction tasks [55,56,57]. To estimate the expected prevalence of MDR strains, Monte Carlo simulations were conducted using 10,000 bootstrap iterations with the boot (v1.3.28) package. Data aggregation and statistical calculations were performed using dplyr (v1.1.0), while simulation distributions were visualized with ggplot2 (v3.4.0). This probabilistic approach has been used in prior studies to model AMR variability and to support risk assessment [56,57]. These methods together allowed us to explore both deterministic and stochastic dimensions of multidrug resistance in the dataset.

Reproducibility: All analyses were performed using open-source R packages, ensuring full reproducibility of the study.

## 5. Conclusions

Our findings confirm the critical state of AMR in Hungarian goose farming, particularly in light of the high prevalence of MDR and XDR *E. coli* strains. The identified resistance patterns and cluster analysis results indicate the presence of strong co-resistance between certain antibiotics, which is likely a consequence of current antibiotic usage practices. Neomycin, amoxicillin, doxycycline, and florfenicol exhibited notably high resistance rates, highlighting the consequences of their widespread application. This situation raises concerns not only for veterinary health but also for public health, as the potential zoonotic transmission of resistant strains could compromise the efficacy of last-resort antibiotics used in human medicine. The One Health approach, which emphasizes the interconnectedness of human, animal, and environmental health, should be considered when developing strategies to combat antimicrobial resistance.

The network analysis confirmed the most significant co-resistance associations, particularly between potentiated sulfonamides and neomycin. The machine learning models used for MDR prediction, including decision trees and PCA-based cluster analysis, proved to be effective tools for identifying antibiotic-resistant strains and mapping resistance trends. Furthermore, Monte Carlo simulations reinforced the high prevalence of MDR strains, emphasizing the necessity for continuous surveillance and stricter antibiotic use regulations.

Overall, these findings underscore the urgent need to reassess antibiotic policies in the poultry sector, with a particular focus on exploring alternative therapeutic options and developing targeted, pharmacologically validated treatment strategies. Future research should aim to provide a deeper understanding of resistance mechanisms, investigate the genetic and ecological factors driving co-resistance, and explore the dynamic relationship between antibiotic use and resistance evolution.

This study highlights the importance of responsible antibiotic use and integrated biosecurity strategies in goose farming, which are essential for mitigating the further spread of antimicrobial resistance.

## Figures and Tables

**Table 1:** Minimum inhibitory concentration (MIC) frequency distribution of *Escherichia coli* isolates (*n* = 91) from geese for antibiotics with established clinical breakpoints. The upper row shows the frequency count, while the lower row represents the percentage of isolates within each MIC category. Red vertical lines indicate clinical breakpoints, while green vertical lines mark epidemiological cut-off values (ECOFF) as defined by European Committee on Antimicrobial Susceptibility Testing (EUCAST).

Antibiotics	Break-Point	0.001	0.002	0.004	0.008	0.016	0.031	0.063	0.125	0.25	0.5	1	2	4	8	16	32	64	128	256	512	1024	MIC_50_	MIC_90_	ECOFF ^3^
	(µg/mL)																								
Amoxicillin	32										2	1	8	14	5	1	0	29	6	3	5	17	64	1024	8
											2.2%	1.1%	8.8%	15.4%	5.5%	1.1%	0.0%	31.9%	6.6%	3.3%	5.5%	18.7%			
Amoxicillin–clavulanic acid ^1^	32					1	0	0	0	0	3	1	6	16	22	18	14	6	1	1	1	1	8	64	8
						1.1%	0.0%	0.0%	0.0%	0.0%	3.3%	1.1%	6.6%	17.6%	24.2%	19.8%	15.4%	6.6%	1.1%	1.1%	1.1%	1.1%			
Ceftriaxone	4				3	10	19	17	12	9	1	3	1	2	1	4	6	1	0	0	0	2	0.06	16	0.125
					3.3%	11.0%	20.9%	18.7%	13.2%	9.9%	1.1%	3.3%	1.1%	2.2%	1.1%	4.4%	6.6%	1.1%	0.0%	0.0%	0.0%	2.2%			
Colistin	2					9	1	7	10	11	16	10	2	2	1	2	15	0	0	0	1	4	0.5	32	2
						9.9%	1.1%	7.7%	11.0%	12.1%	17.6%	11.0%	2.2%	2.2%	1.1%	2.2%	16.5%	0.0%	0.0%	0.0%	1.1%	4.4%			
Doxycycline	16								1	3	0	3	16	12	8	16	20	9	3				16	64	8
									1.1%	3.3%	0.0%	3.3%	17.6%	13.2%	8.8%	17.6%	22.0%	9.9%	3.3%						
Enrofloxacin	2	1	1	0	0	4	12	4	2	4	11	18	9	4	4	3	7	4	2	0	0	1	1	32	0.125
		1.1%	1.1%	0.0%	0.0%	4.4%	13.2%	4.4%	2.2%	4.4%	12.1%	19.8%	9.9%	4.4%	4.4%	3.3%	7.7%	4.4%	2.2%	0.0%	0.0%	1.1%			
Florfenicol	16												1	3	20	24	8	3	13	10	9		16	256	16
													1.1%	3.3%	22.0%	26.4%	8.8%	3.3%	14.3%	11.0%	9.9%				
Imipenem	4							15	9	16	16	29	3	1	1	1							0.5	1	0.5
								16.5%	9.9%	17.6%	17.6%	31.9%	3.3%	1.1%	1.1%	1.1%									
Neomycin	32												1	1	0	10	31	41	4	3			64	64	8
													1.1%	1.1%	0.0%	11.0%	34.1%	45.1%	4.4%	3.3%					
Spectinomycin	128												1	0	0	1	11	59	10	2	4	3	64	128	64
													1.1%	0.0%	0.0%	1.1%	12.1%	64.8%	11.0%	2.2%	4.4%	3.3%			
Potentiated sulphonamide ^2^	4										1	0	6	13	15	16	3	1	2	13	2	19	16	1024	0.5
											1.1%	0.0%	6.6%	14.3%	16.5%	17.6%	3.3%	1.1%	2.2%	14.3%	2.2%	20.9%			

^1^ Ratio 2:1; ^2^ trimethoprim and sulfamethoxazole in a 19:1 ratio, ^3^ epidemiological cut-off values (ECOFF) as defined by the European Committee on Antimicrobial Susceptibility Testing (EUCAST).

## Data Availability

The data presented in this study are available from the corresponding author upon reasonable request.

## Supplementary Materials

The following supporting information can be downloaded at https://www.mdpi.com/article/10.3390/antibiotics14050450/s1. Table S1: Frequency distribution table of minimum inhibitory concentrations (MICs) for *Escherichia coli* isolates (*n* = 91) from ducks, tested against antibiotics without established clinical breakpoints. Figure S1: Proportion of non-wild-type strains per antimicrobial agent based on the epidemiological cutoff values (ECOFF) defined by the European Committee on Antimicrobial Susceptibility Testing (EUCAST). Additional data: Raw Data.

## Abbreviations

The following abbreviations are used in this manuscript:

AMR	Antimicrobial resistance
CAMHB	Cation-adjusted Mueller Hinton Broth
CLSI	Clinical Laboratory Standards Institute
ECOFF	Epidemiological cutoff values
EUCAST	European Committee on Antimicrobial Susceptibility Testing
ESBL	Extended spectrum beta-lactamases
MDR	Multidrug-resistant
MIC	Minimum inhibitory concentration
WHO	World Health Organization
XDR	Extensively drug-resistant

## References

[1] Li H., Chen Y., Machalaba C.C., Tang H., Chmura A.A., Fielder M.D., Daszak P. (2021). Wild Animal and Zoonotic Disease Risk Management and Regulation in China: Examining Gaps and One Health Opportunities in Scope, Mandates, and Monitoring Systems. One Health.

[2] Rahman M.T., Sobur M.A., Islam M.S., Ievy S., Hossain M.J., El Zowalaty M.E., Rahman A.T., Ashour H.M. (2020). Zoonotic Diseases: Etiology, Impact, and Control. Microorganisms.

[3] Poirel L., Madec J.-Y., Lupo A., Schink A.-K., Kieffer N., Nordmann P., Schwarz S. (2018). Antimicrobial Resistance in *Escherichia coli*. Microbiol. Spectr..

[4] Smith O.M., Snyder W.E., Owen J.P. (2020). Are We Overestimating Risk of Enteric Pathogen Spillover from Wild Birds to Humans?. Biol. Rev..

[5] van den Bogaard A.E., Stobberingh E.E. (2000). Epidemiology of Resistance to Antibiotics: Links between Animals and Humans. Int. J. Antimicrob. Agents.

[6] Darwich L., Vidal A., Seminati C., Albamonte A., Casado A., López F., Molina-López R.A., Migura-Garcia L. (2019). High Prevalence and Diversity of Extended-Spectrum β-Lactamase and Emergence of *OXA-48* Producing Enterobacterales in Wildlife in Catalonia. PLoS ONE.

[7] Bodewes R., Kuiken T., Kielian M., Mettenleiter T.C., Roossinck M.J. (2018). Chapter Twelve—Changing Role of Wild Birds in the Epidemiology of Avian Influenza A Viruses. Advances in Virus Research.

[8] Apun K., Kho K.L., Chong Y.L., Hashimatul F.H., Abdullah M.T., Rahman M.A., Lesley M.B., Samuel L. (2010). Detection of *Escherichia coli* O157:H7 in Wildlife from Disturbed Habitats in Sarawak, Malaysia. Res. J. Microbiol..

[9] Benmazouz I., Kövér L., Kardos G. (2024). The Rise of Antimicrobial Resistance in Wild Birds: Potential AMR Sources and Wild Birds as AMR Reservoirs and Disseminators: Literature Review. Magy. Állatorvosok Lapja.

[10] Kovács D., Palkovicsné Pézsa N., Farkas O., Jerzsele Á. (2021). Usage of Antibiotic Alternatives in Pig Farming: Literature Review. Magy. Állatorvosok Lapja.

[11] Essősy M., Fodor I., Ihnáth Z., Karancsi Z., Kovács D., Szalai K.V., Szentmiklósi D., Jerzsele Á. (2020). The Possibilities of Antibiotic-Free Broiler-Hen Fattening, with Special Reference to the Use of Pre- and Probiotics. Magy. Állatorvosok Lapja.

[12] Van Boeckel T.P., Brower C., Gilbert M., Grenfell B.T., Levin S.A., Robinson T.P., Teillant A., Laxminarayan R. (2015). Global Trends in Antimicrobial Use in Food Animals. Proc. Natl. Acad. Sci. USA.

[13] Huang X., Zheng J., Liu C., Liu L., Liu Y., Fan H. (2017). Removal of Antibiotics and Resistance Genes from Swine Wastewater Using Vertical Flow Constructed Wetlands: Effect of Hydraulic Flow Direction and Substrate Type. Chem. Eng. J..

[14] Manyi-Loh C., Mamphweli S., Meyer E., Okoh A. (2018). Antibiotic Use in Agriculture and Its Consequential Resistance in Environmental Sources: Potential Public Health Implications. Molecules.

[15] Qiao M., Ying G.-G., Singer A.C., Zhu Y.-G. (2018). Review of Antibiotic Resistance in China and Its Environment. Environ. Int..

[16] Olasz Á., Jerzsele Á., Balta L., Dobra P.F., Kerek Á. (2023). In Vivo Efficacy of Different Extracts of Propolis in Broiler Salmonellosis. Magy. Állatorvosok Lapja.

[17] Kerek Á., Csanády P., Jerzsele Á. (2022). Antibacterial Efficiency of Propolis—Part 1. Magy. Állatorvosok Lapja.

[18] Kerek Á., Csanády P., Jerzsele Á. (2022). Antiprotozoal and Antifungal Efficiency of Propolis—Part 2. Magy. Állatorvosok Lapja.

[19] Sebők C., Márton R.A., Meckei M., Neogrády Z., Mátis G. (2024). Antimicrobial Peptides as New Tools to Combat Infectious Diseases. Magy. Állatorvosok Lapja.

[20] Hetényi N., Bersényi A., Hullár I. (2024). Physiological Effects of Medium-Chain Fatty Acids and Triglycerides, and Their Potential Use in Poultry and Swine Nutrition: A Literature Review. Magy. Állatorvosok Lapja.

[21] Jócsák G., Schilling-Tóth B., Bartha T., Tóth I., Ondrašovičová S., Kiss D.S. (2025). Metal Nanoparticles—Immersion in the „tiny” World of Medicine. Magy. Állatorvosok Lapja.

[22] Kovács L., Hejel P., Farkas M., László L. (2024). Könyves László Study Report on the Effect of a Litter Treatment Product Containing Bacillus Licheniformis and Zeolite in Male Fattening Turkey Flock. Magy. Állatorvosok Lapja.

[23] Farkas M., Könyves L., Csorba S., Farkas Z., Józwiák Á., Süth M., Kovács L. (2024). Biosecurity Situation of Large-Scale Poultry Farms in Hungary According to the Databases of National Food Chain Safety Office Centre for Disease Control and Biosecurity Audit System of Poultry Product Board of Hungary in the Period of 2021–2022. Magy. Állatorvosok Lapja.

[24] Mag P., Németh K., Somogyi Z., Jerzsele Á. (2023). Antibacterial therapy based on pharmacokinetic/ pharmacodynamic models in small animal medicine-1. Literature review. Magy. Állatorvosok Lapja.

[25] Eda M., Itahashi Y., Kikuchi H., Sun G., Hsu K.-H., Gakuhari T., Yoneda M., Jiang L., Yang G., Nakamura S. (2022). Multiple Lines of Evidence of Early Goose Domestication in a 7,000-y-Old Rice Cultivation Village in the Lower Yangtze River, China. Proc. Natl. Acad. Sci. USA.

[26] Albarella U. (2005). Alternate Fortunes? The Role of Domestic Ducks and Geese from Roman to Medieval Times in Britain. Doc. Archaeobiologiae.

[27] Sun W., Wang D., Yan S., Xue Y. (2022). Characterization of *Escherichia Coli* Strains Isolated from Geese by Detection of Integron-Mediated Antimicrobial Resistance. J. Glob. Antimicrob. Resist..

[28] Yassin A.K., Gong J., Kelly P., Lu G., Guardabassi L., Wei L., Han X., Qiu H., Price S., Cheng D. (2017). Antimicrobial Resistance in Clinical *Escherichia coli* Isolates from Poultry and Livestock, China. PLoS ONE.

[29] Varga C., Guerin M.T., Brash M.L., Slavic D., Boerlin P., Susta L. (2019). Antimicrobial Resistance in Fecal *Escherichia coli* and *Salmonella enterica* Isolates: A Two-Year Prospective Study of Small Poultry Flocks in Ontario, Canada. BMC Vet. Res..

[30] Jeong J., Lee J.-Y., Kang M.-S., Lee H.-J., Kang S.-I., Lee O.-M., Kwon Y.-K., Kim J.-H. (2021). Comparative Characteristics and Zoonotic Potential of Avian Pathogenic *Escherichia coli* (APEC) Isolates from Chicken and Duck in South Korea. Microorganisms.

[31] Afayibo D.J.A., Zhu H., Zhang B., Yao L., Abdelgawad H.A., Tian M., Qi J., Liu Y., Wang S. (2022). Isolation, Molecular Characterization, and Antibiotic Resistance of Avian Pathogenic *Escherichia coli* in Eastern China. Vet. Sci..

[32] Cen D.-J., Sun R.-Y., Mai J.-L., Jiang Y.-W., Wang D., Guo W.-Y., Jiang Q., Zhang H., Zhang J.-F., Zhang R.-M. (2021). Occurrence and Transmission of blaNDM-Carrying Enterobacteriaceae from Geese and the Surrounding Environment on a Commercial Goose Farm. Appl. Environ. Microbiol..

[33] Adiguzel M.C., Baran A., Wu Z., Cengiz S., Dai L., Oz C., Ozmenli E., Goulart D.B., Sahin O. (2021). Prevalence of Colistin Resistance in *Escherichia coli* in Eastern Turkey and Genomic Characterization of an *mcr*-*1* Positive Strain from Retail Chicken Meat. Microb. Drug Resist..

[34] Mitra S., Sultana S.A., Prova S.R., Uddin T.M., Islam F., Das R., Nainu F., Sartini S., Chidambaram K., Alhumaydhi F.A. (2022). Investigating Forthcoming Strategies to Tackle Deadly Superbugs: Current Status and Future Vision. Expert. Rev. Anti Infect. Ther..

[35] McEwen S.A., Collignon P.J. (2018). Antimicrobial Resistance: A One Health Perspective. Microbiol. Spectr..

[36] Collineau L., Bourély C., Rousset L., Berger-Carbonne A., Ploy M.-C., Pulcini C., Colomb-Cotinat M. (2023). Towards One Health Surveillance of Antibiotic Resistance: Characterisation and Mapping of Existing Programmes in Humans, Animals, Food and the Environment in France, 2021. Euro Surveill..

[37] Pulingam T., Parumasivam T., Gazzali A.M., Sulaiman A.M., Chee J.Y., Lakshmanan M., Chin C.F., Sudesh K. (2022). Antimicrobial Resistance: Prevalence, Economic Burden, Mechanisms of Resistance and Strategies to Overcome. Eur. J. Pharm. Sci..

[38] Coculescu B.-I. (2009). Antimicrobial Resistance Induced by Genetic Changes. J. Med. Life.

[39] Whittaker A., Do T.T., Davis M.D.M., Barr J. (2023). AMR Survivors? Chronic Living with Antimicrobial Resistant Infections. Glob. Public Health.

[40] Ferri M., Ranucci E., Romagnoli P., Giaccone V. (2017). Antimicrobial Resistance: A Global Emerging Threat to Public Health Systems. Crit. Rev. Food Sci. Nutr..

[41] Elmberg J., Berg C., Lerner H., Waldenström J., Hessel R. (2017). Potential Disease Transmission from Wild Geese and Swans to Livestock, Poultry and Humans: A Review of the Scientific Literature from a One Health Perspective. Infect. Ecol. Epidemiol..

[42] Tan M.-F., Li H.-Q., Yang Q., Zhang F.-F., Tan J., Zeng Y.-B., Wei Q.-P., Huang J.-N., Wu C.-C., Li N. (2023). Prevalence and Antimicrobial Resistance Profile of Bacterial Pathogens Isolated from Poultry in Jiangxi Province, China from 2020 to 2022. Poult. Sci..

[43] Tasnim Y., Rahman M.K., Abdul-Hamid C., Awosile B. (2025). Beta-Lactamase-Producing *Escherichia coli* in Migratory Geese at West Texas Recreational Parks. Comp. Immunol. Microbiol. Infect. Dis..

[44] EFSA The European Union Summary Report on Antimicrobial Resistance in Zoonotic and Indicator Bacteria from Humans, Animals and Food in 2022–2023. https://www.efsa.europa.eu/en/efsajournal/pub/9237.

[45] Clinical and Laboratory Standards Institute CLSI (2018). Methods for Dilution Antimicrobial Susceptibility Tests for Bacteria That Grow Aerobically.

[46] EUCAST: MIC and Zone Distributions and ECOFFs. https://www.eucast.org/mic_distributions_and_ecoffs/.

[47] Boulianne M., Arsenault J., Daignault D., Archambault M., Letellier A., Dutil L. (2016). Drug Use and Antimicrobial Resistance among *Escherichia coli* and *Enterococcus* spp. Isolates from Chicken and Turkey Flocks Slaughtered in Quebec, Canada. Can. J. Vet. Res..

[48] Lima-Filho J.V., Martins L.V., de Oliveira Nascimento D.C., Ventura R.F., Batista J.E.C., Silva A.F.B., Ralph M.T., Vaz R.V., Rabello C.B.-V., da Silva I.D.M.M. (2013). Zoonotic Potential of Multidrug-Resistant Extraintestinal Pathogenic *Escherichia coli* Obtained from Healthy Poultry Carcasses in Salvador, Brazil. Braz. J. Infect. Dis..

[49] Hesp A., van Schaik G., Wiegel J., Heuvelink A., Mevius D., Veldman K. (2022). Antimicrobial Resistance Monitoring in Commensal and Clinical *Escherichia coli* from Broiler Chickens: Differences and Similarities. Prev. Vet. Med..

[50] Brian V.L. (2019). VET01SEd5 | Performance Standards for Antimicrobial Disk and Dilution Susceptibility Tests for Bacteria Isolated From Animals.

[51] R Core Team R. (2020). A Language and Environment for Statistical Computing.

[52] Kruskal W.H., Wallis W.A. (1952). Use of Ranks in One-Criterion Variance Analysis. J. Am. Stat. Assoc..

[53] Fay M.P., Proschan M.A. (2010). Wilcoxon-Mann-Whitney or t-Test? On Assumptions for Hypothesis Tests and Multiple Interpretations of Decision Rules. Stat. Surv..

[54] Dunn O.J. (1961). Multiple Comparisons among Means. J. Am. Stat. Assoc..

[55] Moradigaravand D., Palm M., Farewell A., Mustonen V., Warringer J., Parts L. (2018). Prediction of Antibiotic Resistance in *Escherichia coli* from Large-Scale Pan-Genome Data. PLoS Comput. Biol..

[56] Yu Y., He Z., Wang C. (2025). Monte Carlo Simulation to Optimize Polymyxin B Dosing Regimens for the Treatment of Gram-Negative Bacteremia. Front. Cell. Infect. Microbiol..

[57] Benkwitz-Bedford S., Palm M., Demirtas T.Y., Mustonen V., Farewell A., Warringer J., Parts L., Moradigaravand D. (2021). Machine Learning Prediction of Resistance to Subinhibitory Antimicrobial Concentrations from *Escherichia coli* Genomes. mSystems.

