# Supplementary material for: Antimicrobial Susceptibility Profiles of Escherichia coli Isolates from Clinical Cases of Geese in Hungary Between 2022 and 2023
Source: Antibiotics (Basel). 2025 Apr 29;14(5):450. doi: 10.3390/antibiotics14050450 (PMC12108276; doi:10.3390/antibiotics14050450)
Supplement: Supplementary file 1 [file antibiotics-14-00450-s001.zip › Supplementary materials.pdf]

Supplementary Table S1 Frequency distribution table of minimum inhibitory concentrations (MICs) for *Escherichia coli* isolates (*n* = 91) from ducks, tested against antibiotics without established clinical breakpoints. The upper row represents the frequency values, while the lower row indicates the corresponding percentage.

**Antibiotics**	**0.001**	**0.002**	**0.004**	**0.008**	**0.016**	**0.031**	**0.063**	**0.125**	**0.25**	**0.5**	**1**	**2**	**4**	**8**	**16**	**32**	**64**	**128**	**256**	**512**	**1024**	**MIC$_{50}$**	**MIC$_{90}$**
		**(μg/mL)**																					
Lincomycin																	2	44	0	7	38	128	1024
																	2.2%	48.4%	0.0%	7.7%	41.8%		
Tylosin																3	0	47	2	23	16	128	1024
																3.3%	0.0%	51.6%	2.2%	25.3%	17.6%		
Tiamulin																	2	62	15	1	11	128	1024
																	2.2%	68.1%	16.5%	1.1%	12.1%		
Vancomycin																		7	20	59	5	512	512
																		7.7%	22.0%	64.8%	5.5%		

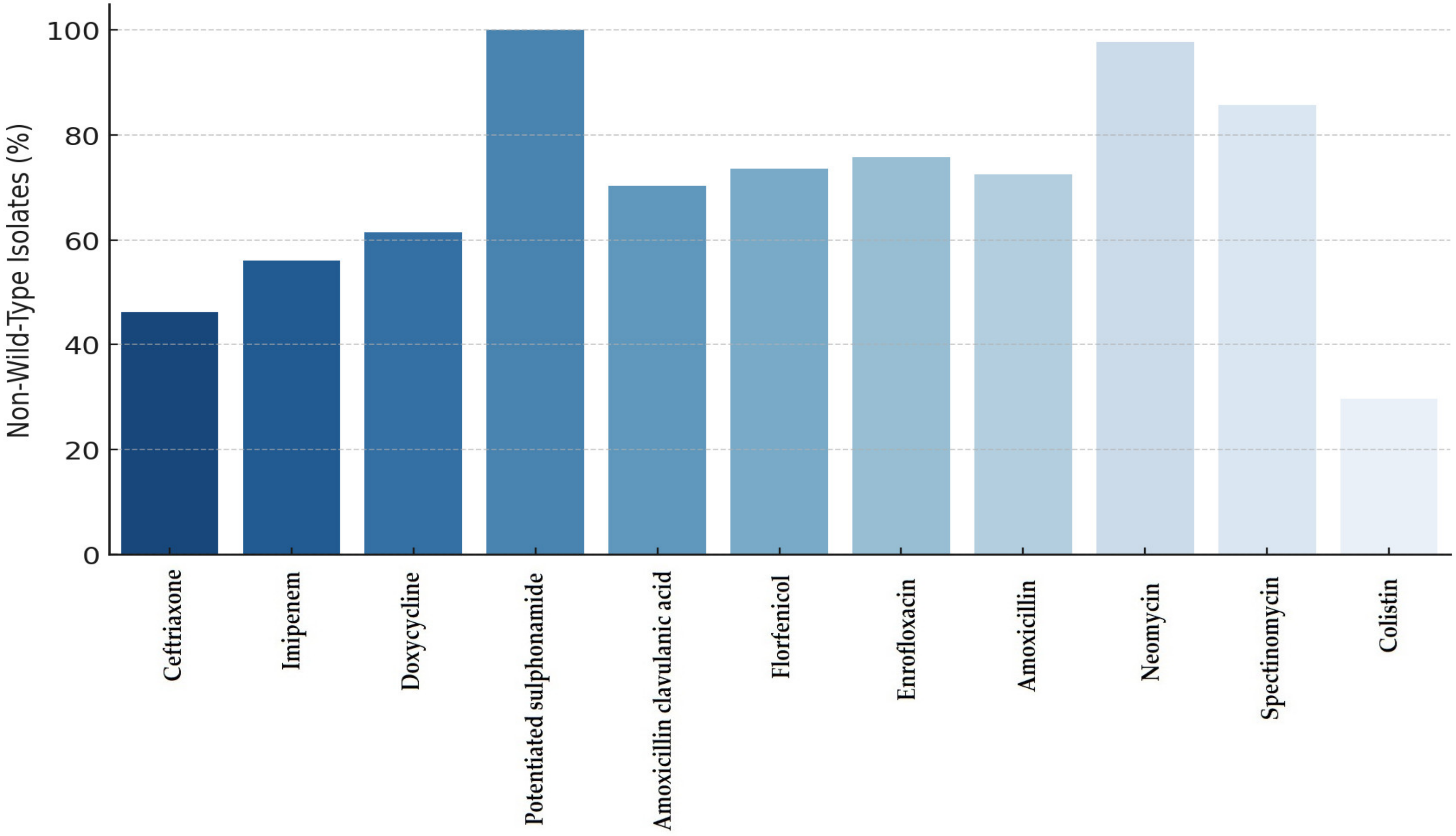

Supplementary Figure S1 Proportion of non-wild-type strains per antimicrobial agent based on the epidemiological cutoff values (ECOFF) defined by the European Committee on Antimicrobial Susceptibility Testing (EUCAST).
